# Birth season affects cow longevity

**DOI:** 10.3168/jdsc.2024-0590

**Published:** 2024-05-31

**Authors:** I.M. Toledo, L. Cattaneo, J.E.P. Santos, G.E. Dahl

**Affiliations:** 1 Department of Animal Sciences, University of Florida, Gainesville, FL 32611; 2 Department of Animal Science, Food and Nutrition (DIANA), Università Cattolica del Sacro Cuore, Piacenza 29122, Italy

## Abstract

Dairy cow longevity is an important economic trait for producers. In modern dairy farming, longevity of dairy cows is the result of culling decisions, which are determined by several risk factors, including diseases, and reproductive and productive performance. Previous studies have documented that seasonal changes affect health, behavior, and performance of dairy cows throughout their life cycle. Increasing cow comfort by making management adjustments to decrease exposure to high temperatures during the hot months gives farmers the opportunity to decrease culling risk factors and possibly increase cow productive life. In the present study, we obtained the records of primiparous and multiparous Holstein cows from Florida (n = 10,812) and California (n = 8,197) during a 10-yr period (2012–2022). We analyzed the relationship between birth season and longevity (i.e., cows that remained in the herd for 5 or more lactations) in Florida (n = 1,567) and in California (n = 1,669). The number of cows dead, sold, the reasons why they were sold, and their relationship with birth season were also analyzed in the Florida dataset. The hypothesis was that birth in a cool season will increase the length of herd productive life and decrease the number of cows sold or dead during all lactations. The birth seasons were cool (CL; cows born in December, January, February, or March) and hot (HS; cows born in June, July, August, or September). Results showed that in Florida, most cows that remained in the herd for more than 5 lactations (i.e., 14.5% of total cow records) were born during CL compared with cows born during HS (1,129, 72% vs. 438, 28%). The same observations were made in California, where greater longevity (i.e., 5 or more lactations, representing 20.4% of total cow records) was attributed to cows born in CL compared with HS cows (939, 56.3% vs. 730, 43.7%). In Florida, a greater number of HS were sold compared with CL cows (765, 52.6% vs. 689, 47.4%). More HS cows were sold due to breeding, foot and leg, and mastitis issues in Florida. Increased cow death during the first 4 lactations was significantly associated with HS (107, 53.8% vs. 92, 46.2%). Results of this study may help farmers create opportunities to make management adjustments related to birth season, or alter negative seasonal factors (i.e., heat stress) to possibly increase cow longevity in dairy herds.

## Highlights

• In Florida and California, the birth of dairy cows during the cool season is associated with increases in the length of productive life to more than 5 lactations.
• Birth season affects the number of cows sold and dead.
• More cows born in Florida during the hottest months are sold due to breeding and foot, leg, and mastitis issues.

## Graphical Abstract

**Figure:**
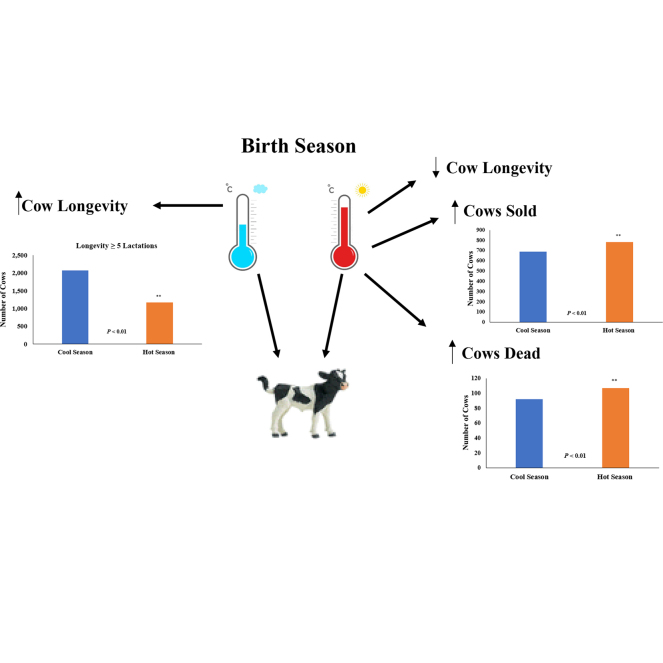
**Summary:** In both Florida and California, the birth of dairy cows during the cool season is associated with increases in the length of productive life to more than 5 lactations. Birth season also affects the number of cows sold and dead. More cows born in Florida during the hottest months are sold due to breeding and foot, leg, and mastitis issues. Birth season adjustments may possibly increase cow longevity in dairy herds.

Longevity of dairy cows is defined as the length of the animal's life or as the length of productive life, which is determined by either culling decisions made by the producer or death of the animal (Schuster et al., 2020). Culling decisions are primarily driven by economic considerations. The capability to achieve high production levels, stay healthy, and reproduce regularly influences the farmer's decision regarding the optimum time to cull a cow. Therefore, dairy replacement management decisions largely determine the average productive life of dairy cows (De Vries, 2020).

In the United States, the productive lifespan of a dairy cow is on average less than 3 lactations, which is much shorter than the natural life expectancy of dairy cows (Pinedo et al., 2010). Cows calve for the first time at 2 yr of age, which brings their total lifespan from birth to death between 4.5 to 6 yr (Vredenberg et al., 2021). In addition to economic consequences, dairy cow longevity is associated with the environmental footprint of the industry and welfare; thus, short productive life has environmental and social consequences, which are inversely associated with dairy sustainability. The ability of dairy farmers to keep their cows for longer could positively enhance the economic performance of the farms by having a higher proportion of mature cows in the higher producing groups, reduce the environmental footprint of the milk industry, and overall help to justify the sustainable use of animals for food production (Brickell and Wathes, 2011; Boulton et al., 2017; Grandl et al., 2019; Dallago et al., 2021).

Previous studies have documented that seasonal changes affect health, behavior, and performance of dairy cows throughout their life cycle. Indeed, the negative effects of heat stress have been extensively studied in lactating and dry dairy cows. The average dairy cow in the United States experiences 96 heat stress days during the year if not cooled (Ferreira et al., 2016). In Florida, on average, dairy farms experience high levels of heat stress for about 267 d, or 73% of the year (Ferreira et al., 2016). Lactating dairy cows start to experience the effects of heat stress when the temperature-humidity index (**THI**) exceeds 68 (Zimbelman et al., 2009). Management adjustments to decrease exposure to high temperatures during the hot months of the year give farmers the opportunity to decrease culling risk factors and possibly increase cow productive life. The physiological and behavioral coping strategies of dairy cows to reduce heat load result in decreases in milk production and are associated with economic losses of about $1.2 billion to the US dairy industry (Key and Sneeringer, 2014). To alleviate the negative effects of heat stress, heat abatement technologies such as shade, fans, soakers, and misters are commonly used for lactating cows on US dairy farms (Spiers et al., 2018).

Heat stress has detrimental effects on milk production, reproductive performance, and immune status of dairy cows during lactation (Hahn, 1999; Kadzere et al., 2002; Collier et al., 2006). Exposure of dry cows to heat stress compromises mammary cell proliferation when dry (Tao et al., 2011) and milk production in the subsequent lactation (do Amaral et al., 2009, 2011; Tao et al., 2011). Furthermore, increased incidence of postpartum disorders such as retained placenta and mastitis has been associated with exposure of cows to high temperatures during the dry period (Thompson and Dahl, 2012). In situations of heat stress, increases in standing time (Nordlund et al., 2019), decreases in activity (West et al., 2003), decreases in rumination time (Soriani et al., 2013), decreases in DMI (Wheelock et al., 2010), and modifications in drinking and eating behaviors of lactating dairy cows are reported. In several studies, cows provided with relief from thermal stress during their last 60 d of pregnancy gave birth to heavier calves, had improved immune status, and produced more milk during the next lactation (do Amaral et al., 2009; Thompson and Dahl, 2012; Tao and Dahl, 2013). There is evidence that adult capacity for milk synthesis may be programmed while calves are still in utero. Calves carried by dams exposed to heat stress during the last 6 wk of gestation produced 19% less milk in their first lactation compared with calves from dams that were cooled during late gestation (Dahl et al., 2017). Further, these effects appear to be transmitted across generations as the lower productivity of heat-stressed dams persists in their daughters and granddaughters (Laporta et al., 2020).

The hypothesis of this study is that birth in a cool season will increase the length of herd productive life and decrease the number of cows sold or dead during all lactations. Our objectives are to better understand the relationship between birth seasons and dairy cow longevity to help farmers create opportunities to make management adjustments to possibly increase productive life in dairy herds.

Records of primiparous and multiparous cows from Florida (n = 10,812) and California (n = 8,197) were obtained from DairyComp (Valley Agricultural Software, California) during a 10-yr period (2012–2022). This study used a data collection approach without direct contact with animal subjects and thus did not require Institutional Animal Care and Use Committee approval. Management of the herds included in the study consisted of best dairy management practices (nutrition, housing, health, and milking practices) in North America. Longevity analysis included Florida (n = 1,567) and California (n = 1,669) cows that were born during either the cool (**CL**; December, January, February, or March) or hot (**HS**; June, July, August, or September) months and remained in the herd for 5 or more lactations (i.e., 5–8 lactations). Available records of CL or HS cows that were either sold (n = 1,454) or died (n = 238) during all lactations were also analyzed. In addition, the relationship between birth season and reasons why cows were sold was analyzed in the Florida cows. Selling reasons included breeding, foot and leg, digestive, and respiratory issues, and mastitis.

The number of cows dead, sold, the reasons why they were sold, and their relationship with birth season were also analyzed in the Florida dataset.

Throughout the whole lactation cycle, primiparous and multiparous cows were housed in freestall barns with a cooling system consisting of shade, soakers, and fans. Monthly temperature (°C) and relative humidity for Florida were obtained from the Florida Automated Weather Network (http://fawn.ifas.ufl.edu/data/reports/). California monthly weather measurements were obtained from the Visual Crossing, weather data services (https://visualcrossing.com/weather/weather-data-services). Temperature-humidity index was calculated based on the following equation described by Dikmen et al. (2008): THI = (1.8 × T + 32) − [(0.55 − 0.0055 × RH) × (1.8 × T − 26)], where T = air temperature (°C) and RH = relative humidity (%).

All statistical analyses were performed using SAS 9.2 (SAS Institute Inc., Cary, NC). The frequency of cows in each season and lactation was analyzed by PROC FREQ of SAS. The *t*-test of SAS was used to compare the means of each group (i.e., cool and hot seasons) to determine significance. PROC MIXED of SAS was used to calculate the THI means.

Previous studies have shown that lactating dairy cows start to experience heat stress and milk production losses when exposed to THI as low as 68 (Collier et al., 2012). In the present study, the estimation of environmental thermal variations due to changes in birth season on dairy cow longevity was determined by THI measurements. Temperature-humidity index measurements were greater during the hot season for both locations (77.0 ± 0.2 in Florida and 73.5 ± 0.2 in California). Cool season THI values were 58.4 ± 0.5 and 52.7 ± 0.5 for Florida and California, respectively. Observation of THI values greater than 68 during the hot season on both locations shows that lactating cows were exposed to significant heat stress during the study period, and potentially subjected to production losses during the hot season.

In Florida, 14.5% (n = 1,567) of the total cow records (n = 10,812) remained in the herd for 5 or more lactations (i.e., lactations 5–8). A significantly greater number of cows born during the coolest months of the year remained in the herd for 5 or more lactations compared with cows born during the hottest months of the year (1,129, 72% vs. 438, 28% cows; *P* < 0.01; Table 1). Similar results were observed for the California data, where 20.4% (n = 1,669) of the total cow records (n = 8,197) remained in the herd for 5 or more lactations. In California, cows born during the cool months had a longer productive life compared with ones born during the hot months (939, 56.3% vs. 730, 43.7% cows; *P* < 0.01; Table 2).

**Table 1:** Distribution of cows with lactation number ≥5 born during either the cool (Dec, Jan, Feb, or Mar) or the hot season (Jun, Jul, Aug, or Sep) in Florida

Lactation number	No. of cows	Birth season	
		Cool season	Hot season
5	968	686	282
6	423	321	102
7	129	96	33
8	47	26	21
Total cows	1,567	1,129 (72%)**	438 (28%)**

** *P* < 0.01.

**Table 2:** Distribution of cows with lactation number ≥5 born during either the cool (Dec, Jan, Feb, or Mar) or the hot season (Jun, Jul, Aug, or Sep) in California

Lactation number	No. of cows	Birth season	
		Cool season	Hot season
5	908	484	424
6	507	318	189
7	204	108	96
8	50	29	21
Total cows	1,669	939 (56.3%)**	730 (43.7%)**

** *P* < 0.01.

Increases in dairy cow productive life associated with birth season may positively affect the dairy industry. When cows have prolonged longevity, rearing costs are lower due to a decrease in the number of replacement heifers and because the rearing investment costs are spread out over a longer productive life. Replacement heifer costs are often ranked as the second or third greatest cost associated with dairy production, just behind feed and labor. The estimated total cost associated with raising a heifer from birth to first calving usually varies between $1,800 to $2,400 per animal (Potts, 2021). On average, it takes around 2 full lactations for the producer to have economic return from a replacement heifer. In addition, exposure to heat stress during late gestation affects the productive life of both the dam and the calf, leading to a shorter productive lifespan (Tao et al., 2019), which may explain the results of the present study, where cows born during the hot months had a shorter productive life compared with those born during the coolest months of the year.

Other benefits associated with longer productive life include reductions in environmental footprint and improvements in welfare and social concerns. It has been documented that by increasing the length of productive life from 2.5 to 4.0 yr, there is a reduction of 9.5% in enteric emissions contribution from replacement heifers (Knapp et al., 2014). Additionally, increases in productive life have been associated with a decrease in methane emission per kilogram of fat- and protein-corrected milk (Grandl et al., 2019), which is linked with a decrease in milk production carbon footprint (Benbrook et al., 2010).

Cow longevity has been an indicator of animal welfare, since longer productive life suggests good health and productivity. Social concerns include early age at culling (Berry, 2015). Thus, animal welfare is important to consumers once it is attached to animal suffering (Molento, 2005). In previous studies, consumers have indicated a willingness to pay more for products obtained from farms with high welfare status (Wolf and Tonsor, 2017). Thus, the demand for greater welfare status by consumers is tightly associated with longer longevity of dairy cows.

Culling is the process of removing animals from the herd due to death, sale, or slaughter (Fetrow et al., 2006). In the present study, Florida cows had a significant greater number of HS sold compared with CL cows, over all lactations (765, 52.6% vs. 689, 47.6%; *P* < 0.01). Increased cow death during the first 4 lactations was significantly associated with HS compared with CL (107, 53.8% vs. 92, 46.2%; *P* < 0.01). Aside from the death of animals, culling decisions are made by the dairy farmer and are influenced by economic interest. Culling can be voluntary, due to low milk production, low milk prices, or adverse behavior, or involuntary, which accounts for the removal of cows from the herd due to injury, reproductive issues, diseases, foot and leg issues, or death (De Vries and Marcondes, 2020).

In the present study, Florida data showed that cows were sold due to breeding, foot and leg, digestive, and respiratory issues, and mastitis. A greater number of HS cows were sold due to breeding issues (458, 53.4% vs. 399, 46.6%; *P* < 0.01; Figure 1a), mastitis (179, 51.6% vs. 168, 48.4%; *P* = 0.02; Figure 1b), and foot and leg issues (71, 58.7% vs. 50, 41.3%; *P* = 0.01; Figure 1c) relative to CL cows. Birth season was not associated with cows sold due to digestive (*P* = 0.52) or respiratory (*P* = 0.57) issues. The risk of culling is not constant over the lifetime of a dairy cow. It changes according to lactation number, stage of lactation, reproductive status, and milk production. Changes in the environment and production needs are also associated with changes in the risk of culling cows (Pinedo et al., 2010; Haine et al., 2017). As the lactation advances, failure to reproduce and lower milk production are the main reasons for increases in culling risk (Pinedo et al., 2010; Haine et al., 2017), whereas injury and death usually are the main reasons for culling in early lactation (Pinedo et al., 2010, 2014). Productive cows that are healthy and have no reproductive and leg or foot issues are favored to stay in the herd longer.

**Figure 1:**
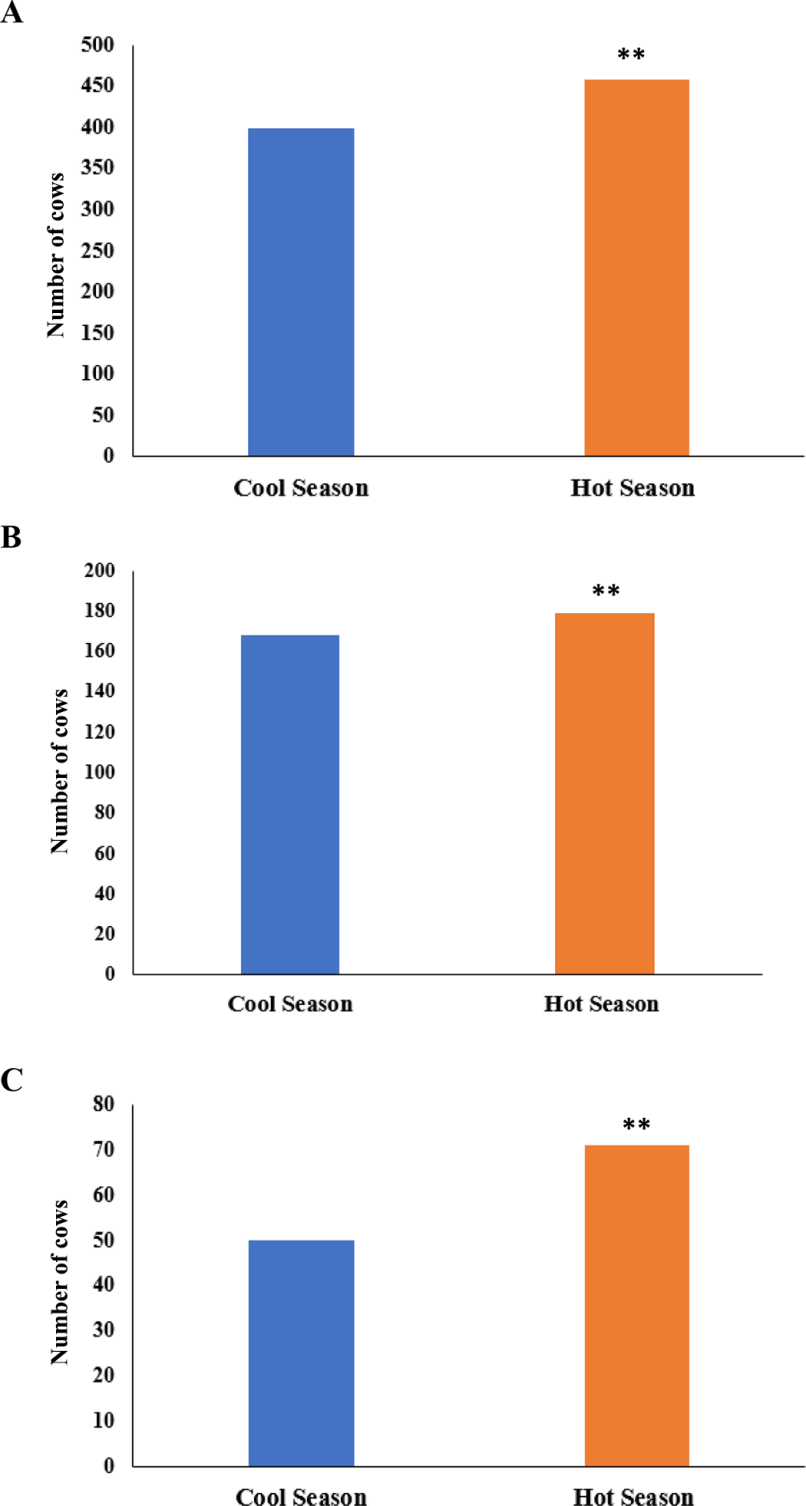
Number of cows born during the cool (Dec, Jan, Feb, or Mar) or hot seasons (Jun, Jul, Aug, or Sep) that were sold because of (A) breeding issues, (B) mastitis, and (C) foot and leg issues. **Indicates a season effect (*P* < 0.01).

In conclusion, the present study showed that, in both Florida and California, the birth of dairy cows during the cool season is associated with increases in the length of productive life to more than 5 lactations. Birth season also affects the number of cows sold and dead. More cows born in Florida during the hottest months were sold due to breeding, foot and leg, and mastitis issues. These results may help producers make birth season adjustments to possibly increase cow longevity in dairy herds.
